# Heat-Shock Proteins Gene Expression in Peripheral Blood Mononuclear Cells as an Indicator of Heat Stress in Beef Calves

**DOI:** 10.3390/ani10050895

**Published:** 2020-05-21

**Authors:** Won-Seob Kim, Jalil Ghassemi Nejad, Sang-Gun Roh, Hong-Gu Lee

**Affiliations:** 1 Department of Animal Science and Technology, Sanghuh College of Life Sciences, Konkuk University, Seoul 05029, Korea; kws9285@hanmail.net (W.-S.K.); jalilgh@konkuk.ac.kr (J.G.N.); 2 Team of An Educational Program for Specialists in Global Animal Science, Brain Korea 21 Plus Project, Sanghuh College of Life Sciences, Konkuk University, Seoul 05029, Korea; 3 Graduate School of Agricultural Science, Tohoku University, Sendai 980-8577, Japan; sanggun.roh@tohoku.ac.jp

**Keywords:** beef calf, heat-shock proteins, heat stress, PBMCs

## Abstract

**Simple Summary:**

This study explores the effects of heat stress on the expression of various heat-shock protein (HSP) genes in bovine peripheral blood mononuclear cells (PBMCs) and cell viability as an indicator of stress in beef calves. We found that heat stress inhibits cell proliferation and increases the expression of HSPs in an in vitro model. In addition, HSPs were found to regulate the physiological mechanisms of adaptation to heat stress in an in vivo model. The results showed that HSPs expression in PBMCs can be used as an indicator of heat stress (HS) in beef calves.

**Abstract:**

This study was conducted to investigate the effect of HS on HSPs gene expression in bovine PBMCs of beef calves in in vitro and in vivo models. In the in vitro experiment, blood samples were collected from the jugular vein of five beef calves (age: 174.2 ± 5.20 days, BW: 145.2 ± 5.21 kg). In the in vivo experiment, sixteen Korean native male beef calves (age: 169.6 ± 4.60 days, BW: 136.9 ± 6.23 kg) were exposed to ambient temperature for seven days (22 to 24 °C, relative humidity 60%; temperature–humidity index (THI) = 68 to 70) and subsequently to the temperature and humidity corresponding to the target THI level for 21 days (HS). For PBMC isolation, blood samples were collected every three days. In the in vitro model, the cell viability was significantly decreased in HS groups compared with the control group (*p* = 0.015). The expression of HSP70 (*p* = 0.022), HSP90 (*p* = 0.003) and HSPB1 (*p* = 0.026) genes was increased in the HS group in in vitro model. In the in vivo experiment, the HSP70 gene expression was increased after sudden exposure to HS conditions (severe THI levels; THI = 88 to 90), whereas HSP90 and HSPB1 showed no differences among the THI groups (*p* > 0.05). However, in the severe THI group, the HSP70 gene expression returned to normal range after six days of continuous HS. In conclusion, the HSP70 gene plays a pivotal role in protecting cells from damage and is sensitive to HS in immune cells compared with other HSP genes in in vitro and in vivo models. In addition, the in vivo models suggest that calves exhibit active physiological mechanisms of adaptation to HS after six days of continuous exposure by regulating the HSP70 gene expression.

## 1. Introduction

Heat stress (HS) alters the body’s physiological mechanisms, resulting in metabolic disorders and decreased immune function in animals [1]. However, severe HS often occurs at the expense of productivity because it interferes with the maintenance of body condition [2]. For instance, heat-stressed animals reduce their dry matter intake (DMI), activity, rumination and metabolic rate to decrease metabolic heat production [3]. HS affects growth performance and energy metabolism by reducing DMI in beef calves [4]. Additionally, HS triggers changes in blood hormones and metabolic indicators by interfering with energy metabolism [5].

Different cellular mechanisms have been proposed to relieve thermal stress in animals [6]. Peripheral blood mononuclear cells (PBMCs) are used to study various stress factors. Among these, a great deal of research is being conducted on the effect of thermal stress on PBMCs. One of the important cell reactions under thermal stress involves transcriptional activation and accumulation of heat-shock proteins (HSPs) [7]. The mechanisms of protective thermoregulation mediated via HSPs during HS are well studied in ruminants [8]. HSPs include a large group of chaperone proteins classified into different families based on molecular size and amino acid sequence similarities [9]. HSPs facilitate adaptation of cells to changes in the environment and play an important role in environmental stress adaptation and heat stress balance, particularly protein function [10]. In addition, HSPs are related to the acquisition of thermotolerance and rapid recovery of heat-induced denatured proteins in their native state [11].

In many previous studies, PBMCs have been used to identify the effects of thermal stress using cell culture systems [12,13]. Thermal stress decreases the viability of PBMCs and increases the expression of genes such as those related to various toll-like receptors, interleukins, cytokine and HSPs [12,14,15]. However, little is known about the tissue-specific responses of these genes in in vivo and in vitro models, particularly in stress events such as HS, which triggers changes in metabolic and endocrine signaling processes, with consequent physiological and behavioral changes. In addition, few studies have investigated the gene expression of HSPs in vivo experiments by identifying the related effects on thermal stress observed in cell cultures of beef calves [16]. Therefore, the objective of this study was to investigate the effects on HSPs gene expression in PBMCs as an indicator of HS in Korean native beef calves using in vivo and in vitro models.

## 2. Materials and Methods

All procedures involving animals were approved by the Institutional Animal Care and Use Committee (IACUC) of Konkuk University (Approval No: KU17125). in vitro and in vivo experiments were completely separate experiments.

### 2.1. In Vitro Experiment

#### 2.1.1. Animals, Sampling and Isolation of PBMCs

Five Korean native male beef calves (age: 174.2 ± 5.20 days, with a BW of 145.2 ± 5.21 kg, non-castrated) under the same management and nutritional regimen were used in this study. Blood was collected in early spring (May 2018) during a thermoneutral period with values of the temperature humidity index consistently below 70, which is considered the upper critical value for cattle [17]. Blood samples (20 mL) were drawn three times during a 2-week period via jugular venipuncture using sodium heparin (10 IU/mL) (BD Biosciences, Billerica, MA, USA) as an anticoagulant to collect whole blood. Immediately after collection, blood samples were stored at room temperature and transferred to the laboratory for blood separation and further analyses. For isolation of PBMCs, the blood samples were processed within 8 h of the sample collection. Density gradient centrifugation was used to separate PBMCs from the whole blood. The whole blood was diluted 1:1 with 1 × PBS (Hyclone, Laboratories, INC., Logan, UT, USA) and layered gently over Histopaque-1077 (Sigma-Aldrich, Inc., St. Louis, MO, USA). All the PBMC isolation steps were performed at room temperature as per manufacturer’s instructions. The isolated PBMCs were washed twice with 1 × PBS. To determine the viability of isolated PBMCs, Trypan blue dye exclusion method [18] was used. The cell pellet obtained was diluted with serum-free RPMI-1640 medium (Sigma-Aldrich, Inc.) and the cell numbers were determined by Hemocytometer (Neubauer-improved, Marienfeld, Germany) using 0.04% Trypan blue (Sigma Life Science, St. Louis, MO, USA). Viability of PBMCs typically exceeded 85% in beef calves. The PBMCs were resuspended at 1 × 10^6^ viable cells/mL in RPMI 1640 medium (Sigma-Aldrich, Inc.) containing 25-m*M* HEPES (Sigma-Aldrich, Inc.) supplemented with heat-inactivated fetal bovine serum (Sigma-Aldrich, Inc.), 2 m*M* l-glutamine (Sigma-Aldrich, Inc.), 100 *U* of penicillin (Sigma-Aldrich, Inc.), 100 μg of streptomycin (Sigma-Aldrich, Inc.) and 0.25 μg of amphotericin B/mL (Sigma-Aldrich, Inc.) in 6-well plates. The time gap until establishment of cultures after blood collections was less than 8 h in each sampling period.

#### 2.1.2. Heat Stress Treatment

The PBMCs isolated from the five Korean native male beef calves were subjected to each treatment in triplicate for 48 h. Initially, all the culture plates were incubated at 37 °C in a humidified CO_2_ incubator (5% CO_2_ and 95% air) for 48 h. After 48 h, cells were exposed to 37 °C continuously (Control; Con) and 42 °C (HS) for 3 h. Con (37 °C) was adopted to imitate thermoneutral conditions and heat treatment (42 °C) was adopted to imitate HS conditions, respectively. After completion of HS, PBMCs were harvested for RNA extraction.

#### 2.1.3. Measurement of Cell Viability (CCK-8 Assay)

Cell proliferation was analyzed using the Cell Counting Kit-8 (CCK-8) according to the manufacturer’s protocol (Dojindo Molecular Technology, Kumamoto, Japan). The cells were seeded 1 × 10^6^ viable cells/mL of medium in a 96-well plate. At the indicated time following treatment (HS), 10 μL CCK-8 solution was added to 90 μL of culture medium. The cells were subsequently incubated for 3 h at 37 °C and the optical density was measured at 450 nm using a microplate reader.

### 2.2. In Vivo Experiment

#### 2.2.1. Animals and Climatic Chamber and Management

Four different experiments were carried out during the study period. Sixteen Korean native male beef calves (average age of all groups: 169.6 ± 4.60 days, with a BW of 136.9 ± 6.23 kg, non-castrated) were assigned equally to one of four treatment groups: threshold (temperature humidity index; THI = 68 to 70, age: 166.5 ± 6.64 days, with a BW of 143.3 ± 16.48 kg), mild (THI = 74 to 76, age: 174.5 ± 2.02 days, with a BW of 133.4 ± 13.47 kg), moderate (THI = 81 to 83, age: 165.3 ± 4.75 days, with a BW of 141.9 ± 13.56 kg) and severe (THI = 88 to 90, age: 172.3 ± 5.75 days, with a BW of 129.1 ± 9.62 kg) stress groups. Each chamber could house two animals at a time; whereas we had a total of four chambers and used two chambers per treatment group. After the completion of two treatments, the other two treatments were carried out using different calves. We performed the first two THI treatments (trial 1; threshold and mild) to have the basic data set regarding the effect of threshold and mild HS. This experiment enabled us to make a better decision whether we should use the next two THI groups (trial 2; moderate and severe) or repeat the first two THI treatment. Thus, we first subjected four calves to the threshold treatment and four other calves to mild HS treatment (two calves per chamber). After finalizing the first two treatments, we conducted the other two treatments, namely, moderate and severe HS treatments, using the same approach, but with different calves. Each animal was provided with an individual supply of feed and water (0800).

The size of each climatic chamber was 2.5 × 2.5 × 3 m^3^ (length, width and height, respectively). The chambers were designed to operate over a temperature range of 18 to 34 °C and a relative humidity (RH) range of 60 to 100%. Each chamber was fitted with controllable temperature and humidity regulators to maintain the desired temperature and RH from 0900 to 1900 h.

This study was conducted to assess the impact of simulated HS conditions in individual climatic chambers on the adaptive capability of Korean native beef calves. The HS conditions were adjusted using the controllable temperature and humidity regulators from 0900 to 1900 h. At all other times (1900 to 0900 h), the temperature and RH were adjusted to match the outside environment (THI 66 to 70). A completely randomized design with four THI treatment groups (threshold, mild, moderate and severe) was used—Calves were subjected to ambient temperature (22 °C) for 7 days (TN) in the adaptation period one week prior to the beginning of the experiment, after which the temperature and humidity in the chambers were raised to each already defined THI level for 21 days (HS). The four THI treatments were defined as threshold (22 to 24 °C, 60% RH: THI = 68 to 70), mild (26 to 28 °C, 60% RH: THI = 74 to 76), moderate (29 to 31 °C, 80% RH: THI = 81 to 83) and severe (32 to 34 °C, 80% RH: THI = 88 to 90) stress levels [19]. Temperature and RH inside the chamber were recorded at intervals of 1 s using two sensors (SHT7×, Sensirion AG, Laubisruetistrasse 508, 712 Staefa ZH, Switzerland). The THI was calculated using the dry bulb temperature (Tdb, °C) and RH using the following formula: THI = (1.8 × Tdb + 32) − [(0.55 − 0.0055 × RH) × (1.8 × Tdb − 26.8)] according to a previous study [20]. The diets used in this study were composed of 40% roughage (*Phleum pratense* L.) and 60% concentrate. The feed was weighed and offered twice daily at 0900 and 1700 h. The chemical composition of the feed is shown in Table 1.

#### 2.2.2. Sampling and Isolation of Peripheral Blood Mononuclear Cells

Blood samples (20 mL) were collected every three days (1100 h) by jugular venipuncture using sodium heparin (10 IU/mL) (BD-Plymouth, PL6 7BP, UK) as an anticoagulant to collect whole blood. PBMCs were isolated from each blood sample using the same methods as in the in vitro experiment. After isolation, PBMCs were used for total RNA extraction.

### 2.3. Total RNA Extraction and Real-Time PCR Analysis

Total RNA was extracted from PBMCs under different treatments using TRIzol™ reagent (Invitrogen, Carlsbad, CA 92018, USA) according to the manufacturer’s instructions. The RNA was measured using an ND-1000 spectrophotometer (Nano Drop Technologies, Wilmington, DE, USA). The A260/280 ratios of all RNA samples were greater than 1.8. The RNA quality was assessed using an RNA 6000 Nano Lab Chip kit (Agilent, Palo Alto, CA, USA). The RNA integrity number (RIN) was confirmed in a Bioanalyzer 2100 (Agilent, Palo Alto, CA, USA) to determine whether the purified total RNA could be used in real-time PCR. The average RIN of PBMCs was 8.2 (7.6 to 9.2). The RNA samples were stored at −70 °C until analysis. First-strand cDNA was synthesized using RNA (1 μg) and an iScript cDNA synthesis kit (Bio-Rad, Hercules, CA, USA) according to the manufacturer’s instructions. The expression of genes in PBMCs including HSP70, HSP90, HSP beta 1 (B1) was analyzed by real-time quantitative PCR (q-PCR) amplifications with SYBR-Green^®^ as described previously [21]. All reactions were performed in triplicate in order to improve the reliability of assessment and in a total reaction volume of 20 μL per well in a 96-well plate using a Chromo4™ four-color real-time detector (MJ Research, Waltham, MA, USA). The reaction mixture contained 100 ng of cDNA, 10 μL of 2× SYBR Green PCR master mix (Bio-Rad) and 0.6 μL of primers at 10 μM (Bioneer, Daejeon, Korea) in autoclaved water. The thermal cycling conditions were as follows: initial incubation at 95 °C for 3 min followed by 40 cycles of denaturation at 95 °C for 10 s, annealing at 60 °C for 30 s and extension at 72 °C for 30 s, after which the samples were heated at 95 °C for 10 s, cooled to 65 °C for 5 s, and then heated to 95 °C at a rate of 0.5 °C/s. The results were monitored using post-PCR melt curve analysis of amplification reactions (in triplicate from all samples) and sequencing amplification products. Primers were designed using the National Center for Biotechnology Information Primer-BLAST (Table 2). The threshold cycles for each sample were normalized to housekeeping genes (GAPDH, RPS15A and B2 M) [22] and the relative expression of the target gene was quantified as the fold change of expression of the target gene relative to the expression of the thermoneutral control according to the 2-ΔΔrCT method [23]. The coefficient of variation of the housekeeping gene was checked prior to the calculation of the results to ensure that it did not exceed 5%.

### 2.4. Statistical Analysis

In the in vitro model, data were reported as least square means ± standard errors of the mean (SEM). The differences in mRNA expression (RT-qPCR) and cell proliferation data between two groups were evaluated using the Student’s *t*-test and the GLM procedure in JMP 5.0 (SAS Institute, Inc., Cary, NC, USA) statistical software. The model included effects of treatment (n = 5) and error term. The calf was used as an experimental unit (n = 5). Three separate sets of in vitro studies with triplicate wells per animal were conducted at 37 °C and 42 °C.

In the in vivo model, data were reported as least squares means ± standard errors of the mean (SEM). To test whether average age and BW of calves in each THI group were statistically different, a statistical analysis was run among the four groups. The mRNA expression (RT-qPCR) of the four groups was evaluated using the repeated-measures analysis and the GLM procedure in JMP 5.0 (SAS Institute, Inc.) statistical software. The model was as follows:

Y*_ijk_* = μ +α*_i_* + β*_j_* + γ(α)*_ik_* + ε*_ijk_*

where Y*_ijk_* is the observation of calf *k* at sampling time *j* for the given treatment *i,* μ is the overall mean, α*_i_* is the fixed effect of treatment *i* (threshold, mild, moderate and severe stress level), β*_j_* is the fixed effect of sampling time *j* (every 3 days), γ(α)*_ik_* is the random effect of calf *k* nested in treatment *i* and ε*_ijk_* is the residual effect. The model included treatments (trial 1; threshold and mild, trial 2; moderate and severe) and calf identifications (age and BW upon entering the chamber) as random variables. The effect of calf nested within treatment was included in the REPEATED statement. A Tukey’s honest significant difference (HSD) test was performed for mean comparisons. The covariance structures (autoregressive order 1, unstructured and compound symmetry) for the repeated measures model were tested and the structure that best fit the model was chosen based on the smallest value of Schwarz’s Bayesian information criterion. The first day of sampling in each THI group was included as a covariate to correct the means and was included in the model when appropriate but was removed from the model when not significant. Differences were considered statistically significant if the *p*-value was less than 0.05. Means with *p*-values between 0.05 and 0.10 reflected a tendency to differ.

## 3. Results & Discussion

Livestock are subjected to various stresses that affect their production, growth performance, reproduction and health. Climate-induced HS is one of the major factors that affects the productivity and adaptability of animals [1]. Beef calves are sensitive to HS, which affects their feed intake and growth performance [24,25]. Moreover, HS also damages calves’ immune system [26,27].

This study was conducted to investigate the effects of HS on various physiological indicators in PBMCs of Korean native beef calves. Prior to an in vivo experiment, the effects of HS on viability and HSPs gene expression were investigated in PBMCs cultured using in an in vitro model.

### 3.1. Proliferation of PBMCs in Cell Culture System

**Figure 1:**
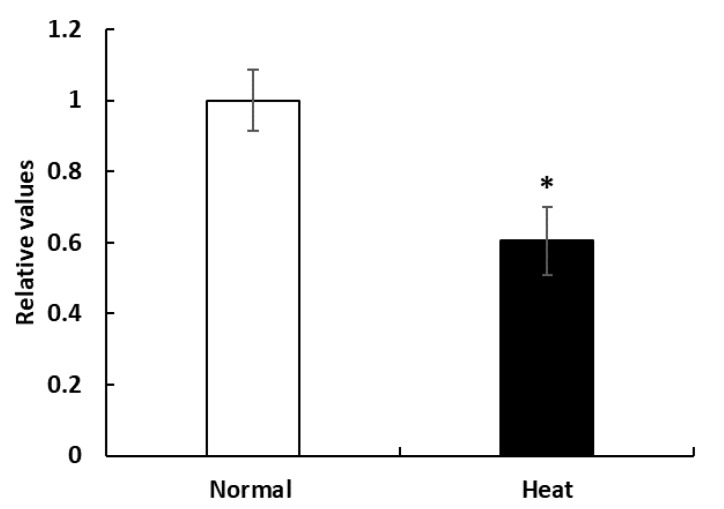
Effects of heat stress on peripheral blood mononuclear cells (PBMCs) proliferation. Cell viability was measured by Cell Counting Kit-8 (CCK-8) assay. Data are presented as the means ± standard errors (n = 5 for each of three experiments). * Means with different superscripts differ significantly (*p* < 0.05) between two groups based on Student’s *t*-test.

To explore the effect of HS on PBMC proliferation, we assayed the cell viability by CCK-8 assay. Figure 1 showed that the cell viability was significantly decreased in the heat-stressed group (42 °C) compared with that of the control group (37 °C) (*p* = 0.015).

HS affects the PBMC morphology and membrane structure and decreases cell viability, and eventually inhibits PBMC proliferation [14]. In the present study, the cell viability was significantly decreased in the HS group (42 °C) compared with that of the control group (37 °C) (*p* = 0.015) (Figure 1). These results are consistent with observations in cattle, in which lower cellular survival was reported after heat shock in PBMCs [5]. Normal conditions (temperature, humidity, CO_2_ and nutrients) during cell culture are critical to cell growth and proliferation, and exposure to abnormal conditions such as HS may induce cell death or inhibit proliferation [12]. The reason why we observed lower cell viability in HS group may be attributed to cell function and activity that was reduced by HS. Furthermore, cell cycle progression in the proliferative responses and sustained proliferative responses are supported by continuous cell division [28] and HS response pathways often evoke cell cycle arrest to avoid the distribution of damaged macromolecules, leading to proliferation inhibition [29]. In addition, HSPs functions can influence the proteins required for cell growth and proliferation in a developmentally regulated manner [30].

### 3.2. The mRNA Expression of HSP70, HSP90 and HSPB1 in PBMCs Cell Culture System and during the Experimental Period in the Climatic Chamber

**Figure 2:**
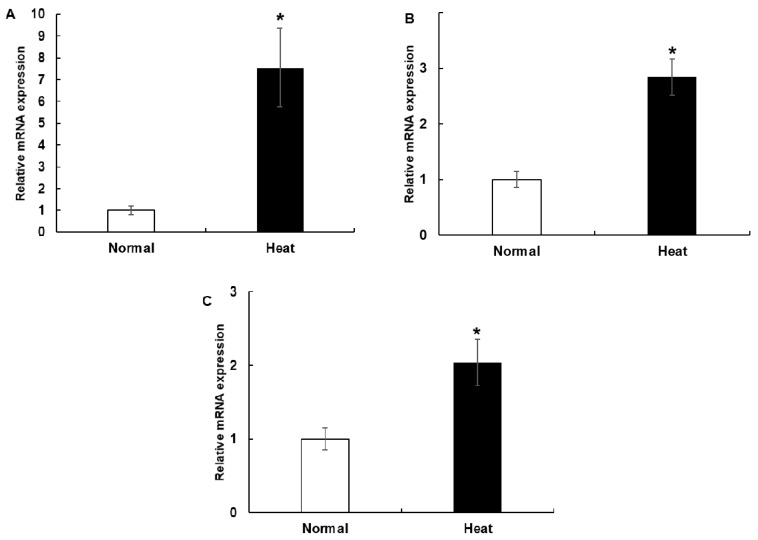
mRNA expression of (**a**), HSP70, (**b**) HSP90, and (**c**) HSPB1 in calf PBMC cultures in an in vitro model. Data are presented as the means ± standard errors (n = 5 for each of three experiments). * Means with different superscripts differ significantly (*p* < 0.05) between two groups based on Student’s *t*-test.

**Figure 3:**
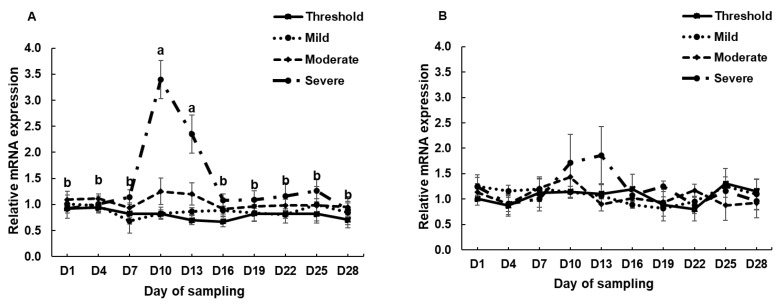

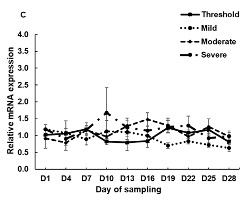
mRNA expression of (**a**), HSP70, (**b**) HSP90, and (**c**) HSPB1 in the PBMCs of calves during the climate chamber experiment. Data are presented as the means ± standards error (n = 4). a, b Means with different superscripts differ significantly (*p* < 0.05) among the sampling dates based on Tukey’s test.

The expression of HSP genes in PBMCs was evaluated in the in vitro experiments (Figure 2). The expression of HSP70 (*p* = 0.022), HSP90 (*p* = 0.003) and HSPB1 (*p* = 0.026) in the heat-stressed PBMCs was significantly higher than in the control group. The expression of HSP genes in PBMCs was evaluated in the in vitro experiments by time course over the total period (28 days; Figure 3). Increased HSP70 was observed after rapid exposure to HS conditions (severe group, *p* = 0.0014). However, as the HS conditions were maintained, HSP70 expression returned to the normal range after 6 days (day 10 to day 16) implying that adaptation occurred.

One possible explanation for higher expression of HSP70 is that the expression of HSP70 likely enhances the protective response to the harmful effects of HS and plays an important role as a cell protection factor for PBMCs in Korean native beef calves. HSP genes code for proteins acting as chaperones protecting cells from HS and facilitate the removal of damaged proteins [31]. In this study, the expression of HSP70 in PBMCs increased significantly when the cells were subjected to HS in in vitro and in vivo models. The HSP70 gene is considered as a reliable biologic marker to quantify HS response in PBMCs [16]. In the present study, the mRNA expression profile of HSP70 in cultured PBMCs is consistent with previous findings involving broilers [32], sheep [33], goats [34] and humans [35]. Sheikh et al. [36] found an increase in HSP70 protein expression in cultured PBMCs when exposed to 42 °C for 3 h of incubation, which is consistent with the results of the present study. In another study, Bhanuprakash et al. [6] found an increase in HSP70 concentration after heat shock at 42 °C for 1 h in PBMCs of cows, which is aligned with our present study. In addition, an increase in HSP70 expression was observed after exposure to heat at 42 °C for 4 h in mammary epithelial cells [14] corroborating this study. As previously reported, the heart rate and rectal temperature as heat-stress indicators increased in each group under the HS conditions (moderate and severe levels) [19,37]. In the current study, expression of the HSP70 gene was increased under rapid exposure to severe HS conditions. The relatively high THI (above the threshold) in the severely stressed group may be another explanation for the elevated expression of HSP70 compared with the threshold group. Among different HSP families, the HSP70 is critically involved in HS response of cells. Therefore, the HSP70 may play an important role in cellular protection against acute HS environmental or physiological stimuli [11]. The expression of HSP90 in PBMCs was significantly increased when the cells were subjected to HS in the in vitro model. In addition, the expression of HSP90 is increased to complement the increased HSP70 expression to protect against HS. HSP90 per se or associated with multi-chaperone complexes, is a major repressor of heat shock transcription factor (HSF1) [8]. In extracts derived from unstressed cells, HSF1 is mainly found as a monomeric polypeptide deficient in specific DNA-binding activity [8]. When cells are stressed, HSF1 homodimerizes, acquires DNA-binding activity and translocates from the cytoplasm to the nucleus [38]. In this study, the increased expression of HSP90 in PBMCs was translated as a repressor of HSF1 to protect against HS. The expression of HSPB1 in PBMCs was significantly increased when the cells were subjected to HS in the in vitro model. HSPB1 mediated different types of stress resistance without organ specificity [39]. HSPB1 is a small HSP involved in many cellular processes and reportedly protects cells against oxidative stress and also rapidly returns to normal levels following the loss of homeostatic challenge [40]. This phenomenon facilitates the overexpression of HSPB1 when cytoprotection is required in HS [41]. In addition, due to changes in phosphorylation and conformation, the HSPB1 alters its response against heat stress [42]. In this study, the HSP90 and HSPB1 genes were found to act as chaperones to protect cells against HS in the in vitro model.

When thermal stress conditions were maintained, the HSP70 gene expression returned to normal range after 3–6 days in in vivo experiments. This result may be explained by homeostasis and adaptation mechanisms in the body at the cellular levels after HS damage. HSP reacts immediately to HS and serves as a chaperone protein [12]. Accordingly, the gene expression of HSP returned to a normal range via adjustment to HS in approximately 6 days in Korean native beef calves. In agreement with the obtained result, in a previous study, the expression of the HSP70 gene was increased after exposure to HS conditions and returned to a normal range after 5 days in Tharparkar cattle [16]. According to this prior study, the HSP70 gene expression returned to the basal level via homeostasis after the lapse of appropriate time, which varies across different breeds. However, the expression of HSP90 and HSPB1 were not altered in our in vivo model (Figure 3b,c). This finding indicates that the HSP70 exhibits more sensitive mechanisms than HSP90 and HSPB1 to facilitate adaptation to HS according to homeostasis regulation.

## 4. Conclusions

In conclusion, the HSP70, HSP90 and HSPB1 genes play an important role in regulating the proliferation of PBMCs in relation to heat stress. In addition, the in vivo model suggests that calves actively carry integrated physiological mechanisms to adapt themselves to heat stress after six days of continuous exposure by regulating the HSP70 gene expression. HSP70 gene regulates physiological homeostasis in the body through heat stress-mediated regulation of cellular activity and proliferation. Results of the present study can be used as a basis for investigations to further improve our understanding of the response of HSPs, especially HSP70 and gene expression in PBMCs as an indicator of heat stress in both in vitro and in vivo models.

## Figures and Tables

**Table 1:** Chemical composition of diets provided to the beef calves.

Item	Basal Diets	
	Concentrate (60%)	Roughage (Timothy Grass) (40%)
% of dry matter		
Crude protein	16.70	6.16
Ether extract	3.13	1.17
Crude fiber	8.84	36.57
Crude ash	6.94	7.49
ADF	10.08	38.69
NDF	22.90	67.13
Calcium	1.43	0.35
Phosphorus	0.50	0.20

ADF = acid detergent fiber; NDF = neutral detergent fiber.

**Table 2:** Primer sequences, lengths and accession numbers.

Gene	Accession Number	Sequence	Length (bp)
HSP70	U09861	F: TACGTGGCCTTCACCGATACR: GTCGTTGATGACGCGGAAAG	171
HSP90	NM_001012670	F: GGAGGATCACTTGGCTGTCAR: GGGATTAGCTCCTCGCAGTT	177
HSPB1	NM_001025569	F: CCTGGACGTCAACCATTCR: GCTTGCCAGTGATCTCCAC	77
GAPDH	NM_001034034.2	F: GGCAAGGTCATCCCTGAGR: GCAGGTCAGATCCACAACAG	166
RPS15A	NM_001037443.2	F: CCGTGCTCCAAAGTCATCGTR: GGGAGCAGGTTATTCTGCCA	200
B2 M	NM_173893.3	F: GACACCCACCAGAAGATGGAR: CAGGTCTGACTGCTCCGATT	125

HSP = heat-shock protein; HSPB1 = heat-shock protein beta 1; GAPDH = glyceraldehyde-3-phosphate dehydrogenase; RPS15A = ribosomal protein S15a; B2M = beta-2-microlobulin.
